# Tissue-based genomic instability markers for predicting malignant transformation in oral leukoplakia and proliferative verrucous leukoplakia: a systematic review

**DOI:** 10.3389/froh.2026.1939172

**Published:** 2026-08-25

**Authors:** Bernadette Quah, Yang Zhang, Rahul Harshad Nagadia, N. Gopalakrishna Iyer, Margaret Sällberg Chen, Nicholas Brian Shannon

**Affiliations:** 1 Department of Oral and Maxillofacial Surgery, Faculty of Dentistry, National University of Singapore, Singapore, Singapore; 2 Department of Laboratory Medicine, Karolinska Institutet, Huddinge, Sweden; 3 Department of Oral and Maxillofacial Surgery, National University Centre for Oral Health, Singapore, Singapore; 4 Department of Head and Neck Surgery, National Cancer Centre Singapore, Singapore, Singapore; 5 Department of Cellular Therapy and Allogeneic Stem Cell Transplantation (CAST), Karolinska University Hospital Huddinge and Karolinska Comprehensive Cancer Center, Stockholm, Sweden

**Keywords:** carcinoma, squamous cell, genetic markers, genomic instability, leukoplakia, oral, oral cancer

## Abstract

**Objectives:**

Although several biomarkers have been described for predicting malignant transformation in oral leukoplakias (OLs) and proliferative verrucous leukoplakias (PVLs), no systematic review has comprehensively evaluated tissue-based genomic instability markers. This review aimed to evaluate the evidence for these markers and their potential role in biomarker panel development.

**Methods:**

A systematic review across PubMed, Embase and Cochrane Library was performed to identify studies evaluating the differences in tissue-based genomic markers between OL and PVL patients with and without malignant transformation.

**Results:**

34 observational studies comprising 3,237 patients were included, and genomic aberrations were categorised into DNA-level, chromosomal, and gene-specific alterations. For studies on OLs, DNA-level and chromosomal markers for which individual studies reported associations with malignant transformation included aneuploidy, impaired DNA repair capacity, loss of heterozygosity, chromosomal instability, and copy number alterations. Multiple gene-specific alterations also showed associations (e.g., TP53, MKI67, FGFR1), but findings varied across studies. The genomic markers of PVLs differed substantially, with fewer consistent predictors found. No meta-analysis was performed as all included studies were observational.

**Conclusions:**

Genomic instability across multiple levels contributes to malignant transformation, and represents a promising biological framework for predicting malignant transformation for OLs. While no single marker reliably demonstrates sufficient predictive performance, the integration of complementary genomic alterations with clinical and histopathological risk factors may provide a basis for the development of robust multi-marker panels. Future prospective studies using standardised detection methods and multivariable prediction models are required before clinical implementation.

**Systematic Review Registration:**

identifier CRD42024585830.

## 1 Introduction

Oral cancer is the sixth most common cancer in the world, with an estimated incidence of more than 370,000 cases and a mortality rate of more than 177,000 individuals annually (1, 2). Oral squamous cell carcinoma (OSCC) accounts for more than 90% of oral cancers (3), and despite advancements in surgery, radiotherapy and chemotherapy alone or in combination, lip and oral cavity cancers remain among the top global causes of death, with the largest number of increases between 2000 and 2023 (4). The 5-year overall survival rate and disease-free survival rates for OSCC have remained at 60% and 50% respectively in recent years, and almost every second patient has postoperative recurrence or metastasis even after radical surgery (5).

The aggressive nature and high mortality associated with OSCC underscore the necessity of early diagnosis and risk stratification. The search for early indicators invariably leads to the study of oral potentially malignant disorders (OPMDs). Oral leukoplakia (OL), one of the most common OPMDs, is defined by the World Health Organisation (WHO) as a predominantly white plaque of questionable risk, having excluded other known diseases that carry no increased risk for cancer (6). It has a worldwide prevalence of 4.11% (7), a malignant transformation rate of 9.8% (95% CI: 7.9–11.7), and a mean evolution time of 3.2 years. While non-homogeneous OL has four times the risk of malignant transformation compared to homogeneous OL (8), the proliferative verrucous leukoplakia (PVL) subtype, defined as a progressive, persistent, and irreversible disorder characterised by the presence of multiple leukoplakias that frequently become warty, is notorious for its increased risk for malignant transformation, with a transformation rate of 45.8% reported (9).

Clinical and demographic risk factors for malignant transformation include lesion size and location, female gender, betel quid or tobacco use, and exposure to nickel (8). Histopathologic assessment by the presence of dysplasia has been considered (10). However, the grading of dysplasia alone may not be entirely sufficient to deduce the risk of malignant transformation. A large cohort study by Hsue et al. found only 4.83% of dysplastic oral leukoplakias (OLs) subsequently progressed to OSCC, and 3.55% among non-dysplastic OLs over a mean follow-up time of 42.6 months (11). Furthermore, the grading of dysplasia is subjective and requires standardisation (6). Hence, the challenge lies especially in the identification of high-risk lesions with little to no dysplasia, with an ever-present clinical dilemma between wide surgical excision and surveillance needs.

Genomic instability represents a fundamental hallmark of cancer and encompasses a wide spectrum of DNA, chromosomal or gene-specific alterations. These alterations collectively reflect progressive genomic derangement preceding malignant transformation, and may provide a foundation for the development of predictive marker panels. While previous reviews have evaluated the role of biomarkers in the malignant transformation of OLs, these reviews have largely investigated a broader range of biomarkers involved in various aspects of the hallmarks of cancer, and different sample types such as tissue, saliva or serum (12–14). Studies on genomic alterations have largely focused on specific parts of genomic instability, such as aneuploidy, loss of heterozygosity or specific genes (15, 16). Consequently, these prior studies provide limited insight into genomic instability as a unified biological process underlying malignant transformation or its potential utility in objective risk prediction.

To our knowledge, no systematic review has comprehensively synthesised tissue-based genomic instability markers specifically evaluated as longitudinal predictors of malignant transformation for oral leukoplakias (OLs) and proliferative verrucous leukoplakias (PVLs). This systematic review therefore aimed to evaluate the available evidence for tissue-based genomic instability markers associated with malignant transformation, and to identify promising candidates for biomarker panel development.

## 2 Materials and methods

This systematic review was conducted in accordance with the Preferred Reporting Items for Systematic Reviews and Meta-analyses (PRISMA) guidelines (17). The review was registered on PROSPERO (CRD42024585830). The review utilised the following PICOTS structure:

- **Population**: Patients histologically or clinically diagnosed with oral leukoplakia (OL) or proliferative verrucous leukoplakia (PVL)
- **Intervention**: Tissue-based genomic instability markers, defined as tissue-derived biomarkers reflecting genomic alterations or molecular events implicated in genomic instability and malignant transformation. This includes markers that directly measure genomic instability (i.e., DNA-level or chromosomal-level markers), and gene-specific alterations that represent downstream molecular consequences of genomic alterations
- **Comparator**: Patients with lesions without genomic instability markers of interest, or with a lower or alternative level of the marker
- **Outcome**: Histologically confirmed malignant transformation
- **Timing:** Minimum of three months duration from initial biopsy to malignant transformation, or last follow-up for lesions without malignant transformation
- **Study Design**: Randomised controlled trials, clinical trials, prospective and retrospective observational studies

### 2.1 Search strategy

A search was performed on 3 September 2024 and updated on 27 September 2025 and 1 August 2026 through the PubMed, Embase, and Cochrane Library databases. The search terms encompassed the following: (1) oral leukoplakia, (2) transformation or progression, and (3) genomic or genetic factors. The full search terms are shown in Supplementary Table S1. From the search results, duplicates were removed, and the articles were then screened by their titles and abstracts, followed by screening of the full-length articles for inclusion on Covidence by two team investigators (B.Q. and Y.Z.).

A manual search of the references of relevant articles was also done, and eligible articles were included. Disagreements were reconciled through discussion and mediation with a third team investigator (N.B.S.) to arrive at a consensus.

### 2.2 Inclusion and exclusion criteria

The following inclusion criteria were used:

- Patients diagnosed with OL or PVL with follow-up and known outcomes of progression or non-progression to oral squamous cell carcinoma
- Analyses of genomic changes for these patients reported
- Full-length articles (randomised controlled trials, clinical trials, prospective and retrospective observational studies) published in English

The following exclusion criteria were applied:

- Patients with leukoplakia outside the oral cavity
- Patients with other potentially malignant disorders outside of oral leukoplakia
- Patients with pre-existing oral cancer at the onset
- Studies with no direct follow-up and reporting of the presence or absence of malignant transformations
- Studies reporting outcomes other than malignant transformation to oral squamous cell carcinoma (e.g., carcinoma *in situ*, severe dysplasia)
- Studies utilising only non-histopathological tissue samples (e.g., blood, saliva)
- Animal studies, *in vitro* studies, Case series, case reports, abstracts, letters to the editor, comments or review articles
- Articles not published in English

The inclusion and exclusion criteria set out to investigate local tissue-specific genomic instability, focusing on markers intrinsic to tissue transformation processes, rather than circulating biomarkers.

### 2.3 Data extraction

Data extraction was performed by two team investigators (B.Q. and Y.Z.). The following information was transferred from the articles onto a data extraction Excel spreadsheet:

- Study characteristics: Author, Year, Study type
- Patient characteristics: Age, Gender, Tobacco use, Alcohol use, Betel quid use,
- Lesion characteristics: Lesion type (OL/PVL), Sample type
- Patient/lesion sample size
- Duration of follow-up
- Genomic aberrations and detection method
- Outcome: Presence or absence of malignant transformation

Discrepancies were discussed and mediated by a third team investigator (N.B.S.) until resolution was achieved before the data was compiled onto a final data extraction Excel spreadsheet. Results were subsequently grouped by the type of genomic markers the study investigated, e.g., DNA ploidy status was classified as a DNA-level alteration, while studies evaluating specific genes were classified as gene-specific alterations.

While a meta-analysis was planned to be done if possible, it was not performed due to the lack of sufficient studies for each domain and the heterogeneity of the studies' methods.

### 2.4 Quality assessment

Two authors (B.Q. and Y.Z.) performed the risk of bias assessment for each study independently. The Newcastle-Ottawa Scale for cohort studies was employed as part of the assessment for risk of bias for observational studies (18), with 7–9 stars being considered Low risk of bias, 4–6 stars considered Unclear risk of bias, and 0–3 stars considered High risk of bias. The Risk of Bias 2 (RoB 2) tool was employed for randomised controlled trials if any were to be included (19). Discrepancies were discussed with a third team investigator (N.B.S.) until a consensus was reached before the final compilation.

## 3 Results

**Figure 1:**
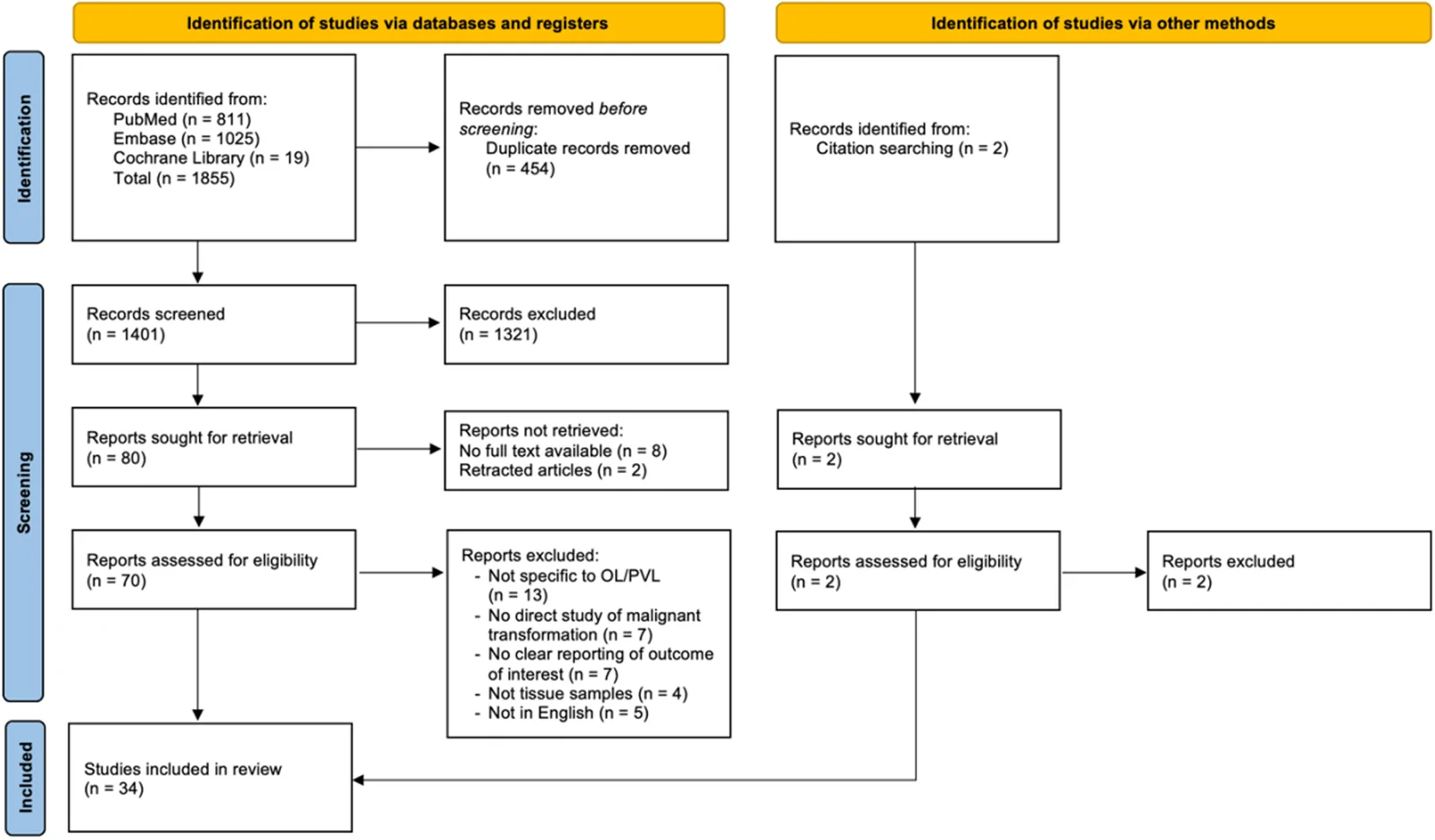
PRISMA flowchart. OL, oral leukoplakia; PVL  , proliferative verrucous leukoplakia.

A total of 1,855 records were identified through the database search, of which 34 studies fulfilled our inclusion criteria and were thus included in the review (Figure 1) (20–53). All studies included were observational, and none were clinical trials. A total of 3,237 patients were included, of which 3,131 patients (30 studies) presented with OL and 106 patients (five studies) presented with PVL. Of these, 622 of the OLs and 52 of the PVLs underwent malignant transformation, translating to a malignant transformation rate of 19.9% and 49.1% respectively, over a reported follow-up duration across studies of 5–300 months. A summary of the included studies' characteristics is shown in Table 1.

**Table 1:** Overview of included studies.

Author	Year	Country	Study Design	Type	Sample Size	Sample Type	Genomic Alteration(s)	Detection Method	Follow-Up (Months)
Qurishi et al.	2025	Saudi Arabia	Prospective	OL	Total: 150 MT: 40 No MT: 110	Fresh frozen	Microsatellite instability, p53, cyclin D1 expression	Not specified	24
Cai et al.	2024	China	Retrospective	OL	Total: 324 MT: 47 No MT: 277	FFPE	CNAs	Whole genome sequencing	67 (34-98)
Mariz et al.	2023	Brazil	Retrospective	OL	Total: 30 MT: 6 No MT: 24	FFPE	FGFR1 expression	IHC, ISH	40.1 (6-168)
Pentenero et al.	2023	Italy	Prospective	OL, PVL	Total (OL): 133 MT: 6 No MT: 127, Total (PVL): 20 MT: 10 No MT: 10	FFPE	DNA ploidy status	Flow cytometry	72
Wils et al.	2023	Netherlands	Retrospective	OL	Total: 89 MT: 25 No MT: 64	FFPE	CNAs, multiple gene expressions (e.g., TP53, NOTCH1)	Next-Generation Sequencing	12-258
Jäwert et al.	2022	Sweden	Retrospective	OL	Total: 28 MT: 14 No MT: 14	FFPE	CNAs	ISH	35 (12-150) (MT group) 102 (50-268) (No MT group)
Monteiro et al.	2022	Portugal	Retrospective	OL	Total: 52 MT: 6 No MT: 46	FFPE	CD44v6, CD147, EGFR, p53, p63, p73, p16, podoplanin expression	IHC	32.4 (2-120)
Li et al.	2021	China	Retrospective	OL	Total: 477^a^ MT: 19 No MT: 458	FFPE	CNAs	Whole genome sequencing	NR
Liu et al.	2021	China	Retrospective	OL	Total: 60 MT: 5 No MT: 55	FFPE	LOLA1 expression	qRT-PCR	38.5
Lv et al.	2020	China	Retrospective	OL	Total: 79 MT: 30 No MT: 49	FFPE	PTHLH expression	IHC	5-75
Thomas et al.	2020	India	Prospective	OL	Total: 181 MT: 12 No MT: 169	FFPE	DNA ploidy status, DNA telomerase activity, DNA repair capacity	Flow cytometry, TRAP, MSA	102
Baran et al.	2019	Germany	Retrospective	OL	Total: 135 MT: 53 No MT: 82	FFPE	MAGE-A expression	IHC, RT-PCR	17 (MT group), 153.5 (no MT group)
Farah et al.	2019	Australia	Retrospective	OL	Total: 13 MT: 5 No MT: 8	FFPE	Genomic mutations pathways	Whole exome sequencing, qPCR, IHC	16-220
Ding et al.	2018	China	Retrospective	OL	Total: 78 MT: 19 No MT: 59	FFPE	Notch1 expression	IHC	74.18
Upadhyaya et al.	2018	USA	Retrospective	PVL	Total: 20 MT: 9 No MT: 11	FFPE	p16INK4A, p53 expression	IHC, ISH	91.86
Wu et al.	2018	China	Retrospective	OL	Total: 98 MT: 21 No MT: 77	FFPE	TGM3 expression	IHC, qPCR	57 (13-173) (MT group) 95 (20-240) (no MT group)
Habiba et al.	2017	Japan	Retrospective	OL	Total: 79 MT: 37 No MT: 42	FFPE	ALDH1, podoplanin expression	IHC	42.1 ± 34.1
Sakata et al.	2017	Japan	Retrospective	OL	Total: 150 MT: 23 No MT: 127	FFPE	SMAD4 expression	IHC	34
Zhang et al.	2017	South Korea	Retrospective	OL	Total: 154 MT: 22 No MT: 132	FFPE	Axin2 and SNAI1 expression	IHC	130
Kil et al.	2016	South Korea	Retrospective	OL	Total: 27 MT: 7 No MT: 20	FFPE	CNVs	RT-PCR	43 ± 36.7 (MT group) 117.68 ± 28.8 (no MT group)
Gouvêa et al.	2013	Brazil	Retrospective	PVL	Total: 7^a^ MT: 5 No MT: 2	FFPE	DNA ploidy status, Ki67, Mcm2 and geminin expression	Image cytometry, IHC	88.62 ± 82.26
Graveland et al.	2013	Netherlands	Retrospective	OL	Total: 43 MT: 6 No MT: 37	FFPE	LOH, p53 expression	IHC, qPCR	20.3 (11-31) (MT group)
Liu et al.	2013	China	Retrospective	OL	Total: 141 MT: 37 No MT: 104	FFPE	ALDH1 and CD133 expression	IHC	61.2 ± 43.2 (MT group) 67.2 ± 45.6 (no MT group)
Ries et al.	2013	Germany	Retrospective	OL	Total: 98 MT: 53 No MT: 45	FFPE	EGFR expression	IHC	60
Siebers et al.	2013	Netherlands	Retrospective	OL	Total: 102 MT: 16 No MT: 86	FFPE	Chromosomal instability	Image cytometry, FISH	27 (MT group) 91.5 (no MT group)
Ries et al.	2012	Germany	Retrospective	OL	Total: 74 MT: 24 No MT: 50	FFPE	MAGE-A expression	qPCR	60
Bremmer et al.	2011	Netherlands	Retrospective	OL	Total: 62 MT: 13 No MT: 49	FFPE	DNA ploidy status	Image cytometry	69 (10-193)
Cao et al.	2011	China	Retrospective	OL	Total: 76 MT: 37 No MT: 39	FFPE	EZH2 expression	IHC	24 (MT group) 136.9 (no MT group)
Matsubara et al.	2011	Japan	Retrospective	OL	Total: 112 MT: 6 No MT: 106	FFPE	*Δ*Np63 expression	IHC	NR
Klanrit et al.	2007	United Kingdom	Retrospective	PVL	Total: 6 MT: 6 No MT: 0	FFPE	DNA ploidy status	Image cytometry	144-300
Mogi et al.	2003	Japan	Retrospective	OL	Total: 60 MT: 13 No MT: 47	FFPE	p53 expression	IHC	28.6 (6-84) (MT group) 26.1 (3-90) (no MT group)
Nogami et al.	2003	Japan	Retrospective	OL	Total: 13 MT: 7 No MT: 6	FFPE	p53, Bcl-2, Bax expression, Ki-67	IHC	60
Jiang et al.	2001	Japan	Retrospective	OL	Total: 13 MT: 13 No MT: 0	FFPE	LOH, microsatellite instability, fractional allelic loss	PCR	17.7 (2-87)
Chang et al.	2000	Taiwan	Retrospective	PVL	Total: 53 MT: 22 No MT: 31	FFPE	p53, p21WAF1 expression	IHC	42

MT, malignant transformation; FFPE  , formalin-fixed paraffin-embedded tissue samples; CNAs  , copy number alterations; IHC, immunohistochemistry; ISH, *in situ* hybridisation; qRT-PCR, quantitative reverse transcription polymerase chain reaction; TRAP, Telomeric Repeat Amplification Protocol (TRAP); MSA, mutagen sensitivity assay; qPCR, quantitative or real-time polymerase chain reaction; CNVs, copy number variations; LOH, loss of heterozygosity.

a Only samples with clear information on the presence or absence of malignant transformation were included.

To align with the multifaceted nature of genomic instability, the genomic changes are classified into three major domains: DNA-level alterations, chromosomal alterations and gene-specific alterations. Details of these genomic alterations observed in the included cohorts are presented in Tables 2–4 for OL and Table 5 for PVL.

**Table 2:** Summary of DNA-level alteration findings for oral leukoplakia.

Study	Variable	Summary of Findings			
Pentenero et al.	DNA ploidy status		MT	No MT	*p*-value
		Aneuploidy presence	4/6 (66.7%)	33/127 (26%)	0.03
		High aneuploidy	2/6 (33.3%)	5/127 (3.9%)	0.002
		PPV	10.8% (6−18.6)		
		NPV of diploid status	97.9% (93.8–99.3)		
Thomas et al.	DNA ploidy status		MT	No MT	*p*-value
		Aneuploidy	8/12 (66.7%)	37/157 (23.6%)	0.0035
		Sensitivity	66.67% (34.89–90.08)		
		Specificity	77.30% (70.10–83.49)		
		PLR	2.94 (1.80–4.80)		
		NLR	0.43 (0.19–0.96)		
	DNA telomerase activity		MT	No MT	*p*-value
		Telomerase positive	10/12 (83%)	91/169 (53.8%)	0.0654
		Sensitivity	83.33% (51.59–97.9)		
		Specificity	46.15% (38.47–53.98)		
		PLR	1.55 (1.16–2.07)		
		NLR	0.36 (0.10–1.29)		
	DNA repair capacity		MT	No MT	*p*-value
		Hypersensitivity	11/12 (91.6%)	27/169 (15.9%)	0.0001
		Sensitivity	91.67% (61.52 −99.79)		
		Specificity	84.02% (77.61–89.20)		
		PLR	5.74 (3.90–8.44)		
		NLR	0.1 (0.02–0.65)		
Farah et al.	Gene mutation pathways	DNA damage repair pathways were in the top 10 significantly enriched pathways for progressive lesions e.g., mismatch repair pathway, BRCA pathway, Fanconi anaemia pathway			
Bremmer et al.	DNA ploidy status		MT	No MT	*p*-value
		Abnormal DNA content	7/13 (53.8%)	20/49 (40.8%)	
		Aneuploidy	HR of 3.7 (1.1–13.0)		0.039
		Sensitivity	54%		
		Specificity	59%		
		PPV	26%		
		NPV	83%		

MT, malignant transformation; PPV, positive predictive value; NPV, negative predictive value; PLR, positive likelihood ratio; NLR, negative likelihood ratio.

**Table 3:** Summary of chromosome-level alteration findings for oral leukoplakia.

Study	Variable	Summary of Key Findings		
Graveland et al.	LOH	- All OLs with MT showed LOH at 9p - LOH at 9p sig. related to increased risk of MT (*p* = 0.014)		
Jiang et al.	LOH	- Sig. difference in LOH at 5q21–23 before and after MT (*p* = 0.0137) - Other loci with high incidence in OL with MT: 5q21–23, 6p25–27, 6q21–23, and 11q23		
	FAL	- Mean value of FAL of 0.46 for OL with MT, 0.27 without MT - Sig. difference in mean FAL with and without MT (*p* < 0.05)		
	MI	- No OLs showed high level MI - MI observed after MT in 2 OLs where not present before MT		
Qurishi et al.	MI	- 10/25 (40%) OL with MT were tested positive for MI, compared to 25/150 (16.7%) without MT - Sig. association between expression of MI + p53 + cyclin D1 and MT (*p* < 0.05)		
Siebers et al.	Chromosomal instability	- Chromosomal instability was a stronger marker for MT - Hazard ratio of 7.2 with image cytometry (*p* < 0.001) - Hazard ratio of 6.8 with fluorescence *in situ* hybridisation (*p* < 0.001)		
Cai et al.	CNAs	Results	- CNA score sig. higher in MT group (*p* < 0.001) - CNA score predictive of MT (*p* < 0.001)	
		Significant regions	Gain	Chromosome 3, 8, 20
			Loss	Chromosome 3, 5, 8, 9, 13
Wils et al.	CNAs	Results	- Presence of CNA not sig. associated with the risk of MT (*p* = 0.313) - Number of CNAs sig. associated with the risk of MT (*p* = 0.011)	
		Significant regions	Gain	20p11
			Loss	3p14, 8p23, 13q12
Jäwert et al.	CNAs	- Reported CNAs specific to genes - CNAs most common in EGFR (43.5%), followed by CDKN2A (26.1%), CCND1 (26.1%), and MYC (8.3%) - No sig. difference in loss of CDKN2A (*p* = 0.16)		
Li et al.	CNAs	- More CNAs in MT group compared to no MT (*p* < 0.0001) - Sig. higher CNA score and frequency in OLs with MT (*p* < 0.001) - High occurrence rates at Chromosomes 1, 2, 7, 8, 20		
Kil et al.	Copy number variations	Results	- 6/6 (100%) of OL with MT had CNVs, compared to 6/17 (35.3%) of OL without MT (*p* = 0.014) - 4/6 (66.7%) of OL with MT had >3 CNVs, compared to 1/17 (5.9%) without MT (*p* = 0.008) - CNV outside 3p rarely seen in OL without MT	
		Significant regions	- 9p21 (*p* = 0.04) - 13q14 (*p* = 0.011)	

MT, malignant transformation; LOH, loss of heterozygosity; FAL, fractional allelic loss; MI, microsatellite instability; CNAs, copy number alterations.

**Table 4:** Summary of gene-specific alteration findings for oral leukoplakia.

Study	Gene	Protein	Significant Findings
P53 Family			
Qurishi et al.	TP53	p53	18/30 (60%) p53-positive OLs had MT Sig. association between expression of MI + p53 + cyclin D1 and MT (*p* < 0.05)
Wils et al.	TP53	p53	TP53 mutation sig. associated with MT (HR 2.70, *p* = 0.013)
Monteiro et al.	TP53	p53	No sig. relationship with MT-free survival (*p* = 0.574–0.642)
	TP63	p63	No sig. relationship with MT-free survival (*p* = 0.478–0.745)
	TP73	p73	No sig. relationship with MT-free survival (*p* = 0.526–0.582)
Graveland et al.	TP53	p53	P53 mutation by sequencing sig. associated with MT (*p* = 0.009) ositive P53 IHC or P53 field not sig. related to MT-free survival
Matsubara et al.	TP63	*Δ*Np63 isoform	*Δ*Np63-labelling index of OL with MT was sig. higher than without MT (49.3 vs. 34.2%, *p* = 0.0035)
Mogi et al.	TP53	p53	P53 positive rates in OL with MT sig. higher than without MT (77 vs. 42%, *p* = 0.0120)
Nogami et al.	TP53	p53	Many p53 positive cells seen in OL with MT, compared to a few p53 positive cells in OL without MT
Cell Cycle Regulators			
Qurishi et al.	CCND1	Cyclin D1	22/45 (48.9%) cyclin D1-positive OLs had MT Sig. association between expression of MI + p53 + cyclin D1 and MT (*p* < 0.05)
Wils et al.	CDKN2A	p16	CDKN2A mutations + loss of 9p21 were not significantly associated with MT
Monteiro et al.	CDKN2A	p16	No sig. relationship with MT-free survival (*p* = 0.081–0.126)
	MKI67	Ki-67	Sig. higher positive Ki-67 expression in OL with MT (14.9 vs. 6.4%, *p* < 0.05)
Mariz et al.	FGFR1	FGFR1	All OL with MT has high FGFR1 expression (*p* = 0.006) Higher risk of MT in OL with high FGFR1 expression (HR 7.3, *p* = 0.016)
Cao et al.	EZH2	EZH2	EZH2 expression strongly associated with MT-free survival in an expression level-dependent manner (*p* < 0.0001)
Apoptosis Regulators			
Wils et al.	CASP8	Caspase-8	Sig. association with higher MT rate (HR 16.69, *p* = 0.031) and shorter MT-free survival (*p* = 0.010)
Nogami et al.	BCL2	Bcl-2	Weak-moderate Bcl-2 immunoreactivity in OL with MT, vs. weak immunoreactivity in OL without MT
	BAX	Bax	Weak Bax positivity in OL with MT, vs. moderate to strong positivity in OL without MT
Oncogenic Signalling Pathways			
Monteiro et al.	EGFR	EGFR	No sig. relationship with MT-free survival (*p* = 0.461–0.610)
Ries et al.	EGFR	EGFR	Sig. difference in EGFR expression rate in OL with and without MT (*p* = 0.017) Odds ratio 4.83 for EGFR over 44.96
Wils et al.	NOTCH1	Notch1	Associated with a longer MT-free survival, but not sig. (*p* = 0.149)
Ding et al.	NOTCH1	Notch1	Sig. decreased expression in OL with MT (*p* = 0.001)
Sakata et al.	SMAD4	Smad4	Low expression sig. associated with MT (*p* = 0.0017), sig. predictor for MT (HR 2.63, *p* = 0.043)
Zhang et al.	AXIN2	Axin-2	High expression sig. associated with MT in multivariate analysis (HR 7.47, *p* = 0.001)
Cancer Stem Cell, Differentiation and Invasion Markers			
Habiba et al.	Podoplanin	Podoplanin	Sig. higher incidence of expression in OL with MT (84 vs. 52%, *p* = 0.003) Expression sig. associated with MT (HR = 2.95, *p* = 0.007 in univariate, HR 2.62, *p* = 0.039 in multivariate)
	ALDH1	ALDH1	Sig. higher incidence of expression in OL with MT (73 vs. 50%, *p* = 0.037) Expression sig. associated with MT (HR = 2.91, *p* = 0.005 in univariate, HR 3.02, *p* = 0.004 in multivariate)
Monteiro et al.	Podoplanin	Podoplanin	Higher expression had sig. association with MT (*p* < 0.001)
	CD44	CD44v6	No sig. relationship with MT-free survival (*p* = 0.510–0.803)
Liu et al.	CD133	Prominin-1	Sig. higher incidence of expression in OL with MT (51.4 vs. 12.5%, *p* < 0.001) Sig. higher number of CD133-positive OL had MT (59.4 vs. 16.5%, *p* < 0.001)
	ALDH1	ALDH1	Sig. higher incidence of expression in OL with MT (70.3 vs. 26.9%, *p* < 0.001) Sig. higher number of ALDH1-positive OL had MT (48.1 vs. 12.6%, *p* < 0.001)
Zhang et al.	SNAI1	Snail	Sig. higher incidence of MT in Snail-positive (*p* = 0.007) and high expression OL (*p* < 0.001) Snail expression sig. associated with MT (HR 3.28, *p* = 0.007 in univariate, HR 4.41, *p* = 0.001 in multivariate)
Wu et al.	TGM3	TGase3	Low levels sig. associated with increased risk of MT (*p* = 0.0005) TGM3 expression is an independent predictor for MT (HR 5.045, *p* = 0.004)
Immune and Tumour Microenvironment			
Baran et al.	MAGE-A	MAGE-A	Sig. higher positivity in OL with MT with IHC (62.5 vs. 3.5%, *p* = 0.001) PPV of 93%, NPV of 74.3%, MAGE-A is a highly reliable predictor for MT (*p* < 0.001)
Ries et al.	MAGE-A	MAGE-A	Sig. higher positivity in OL with MT (46 vs. 0%, *p* = 0.00001)
Lv et al.	PTHLH	PTHrP	PTHrP expression levels sig. associated with MT (HR 4.54, *p* = 0.000 in univariate, HR 3.3, *p* = 0.001 in multivariate)
Monteiro et al.	BSG	CD147	No sig. relationship with MT-free survival (*p* = 0.586–0.779)
Others			
Wils et al.	NSD1	NSD1	Mutation sig. associated with longer MT-free survival
Liu et al.	LOLA1	LOLA1 lncRNA	Expression sig. higher in OL with MT (*p* = 0.003–0.058) Sig. higher number of OL with LOLA1 expression had MT (44.4 vs. 5.6%, *p* = 0.017)

MT, malignant transformation; PPV, positive predictive value; NPV, negative predictive value; HR, hazard ratio.

**Table 5:** Summary of genomic alteration findings for proliferative verrucous leukoplakia.

Study	Variable	Key Findings	
DNA-Level Alterations			
Gouvêa et al.	DNA ploidy status	Aneuploidy presence	4/5 (80%) in PVL with MT, 1/2 (50%) in PVL without MT
Klanrit et al.	DNA ploidy status	Aneuploidy presence	5/6 (83.3%) of PVL with MT had aneuploidy prior to MT
Pentenero et al.	DNA ploidy status	Aneuploidy presence	No sig. association (*p* = 0.329)
		High aneuploidy	No sig. association (*p* = 0.305)
Gene-Specific Alterations			
Chang et al.	CDKN1A (p21)	Sig. higher MT in p21 positive OL (80 vs. 32%, *p* = 0.002)	
	TP53 (p53)	Sig. higher MT in p53 positive OL (93 vs. 42%, *p* = 0.00008)	
Upadhyaya et al.	TP53 (p53)	<25% stained positive for p53	
	CDKN2A (p16)	No sig. association between p16 positivity and MT (*p* = 0.658)	
Gouvêa et al.	MKI67 (Ki-67)	No sig. correlation	
	MCM2 (Mcm2)	Higher MCM2 expression sig. correlated with increasingly severe epithelial changes (*p* = 0.03)	
	GMNN (geminin)	No sig. correlation	

MT, malignant transformation.

### 3.1 DNA-level alterations

Four studies involving 389 patients investigated the role of DNA-level alterations in the malignant transformation of OL (Table 2) (23, 30, 32, 46). Three studies investigated DNA ploidy status, all of which reported significant associations between aneuploidy and malignant transformation (23, 30, 46). The studies reported the presence of aneuploidy to have a poor to fair sensitivity of 54%–66.7%, specificity of 59%–77.3%, a positive predictive value (PPV) of 10.8%–26%, and a notably high negative predictive value of 83%–97.9%.

Two studies evaluated the role of damage repair capacity and pathways in the malignant transformation of OL (30, 32). Thomas et al. quantified DNA repair capacity using a bleomycin-induced Mutagen Sensitivity Assay (MSA) and reported a significantly increased proportion of hypersensitivity to bleomycin in OL with malignant transformation (*p* = 0.0001), with a sensitivity of 91.7% and a specificity of 84.0%. Farah et al. similarly found that DNA damage repair-related pathways were some of the most significantly affected pathways in patients with progressive OL.

Thomas et al. also investigated DNA telomerase activity. While a higher proportion of telomerase positivity was seen in progressive OL, the difference was not significant (*p* = 0.065). The authors also reported a sensitivity of 83.3% and a specificity of 46.2% for DNA telomerase activity.

### 3.2 Chromosomal alterations

Nine studies comprising 1,253 patients evaluated the role of structural aberrations at the chromosomal level in OL progression (Table 3) (20, 21, 24, 25, 27, 39, 41, 44, 52). These included loss of heterozygosity (LOH), microsatellite instability, chromosomal instability, fractional allelic loss, and copy number alterations/variations. LOH was evaluated in two studies (41, 52); Graveland et al. identified LOH at chromosome 9p to be significantly associated with malignant transformation, while Jiang et al. found LOH at 5q21–23 to be significantly different between progressive and non-progressive lesions. Jiang et al. also reported significant differences in mean fractional allelic loss between the two groups (*p* < 0.05).

Two studies evaluated the role of microsatellite instability (20, 52). Jiang et al. did not report high levels of microsatellite instability in any progressive OLs. Qurishi et al. found a higher proportion of patients with microsatellite instability in progressive lesions; although statistical analyses showed a significant association with malignant transformation, these analyses were in combination with other markers (p53 and cyclin D1). Chromosomal instability was studied by Siebers et al. who reported it as a strong marker of malignant transformation, with hazard ratios of 6.8–7.2 (44).

Copy number alterations (CNAs) and variations (CNVs) were investigated by five studies (21, 24, 25, 27, 39). Cai et al., Wils et al., Li et al. and Kil et al. reported significant associations between the number of CNAs/CNVs and CNA score and malignant transformation. The locations of these CNAs were highly variable between studies. Cai et al. and Wils et al. both reported significant copy number gains at chromosome 20, and losses at chromosomes 3, 8 and 13. Li et al. and Kil et al. reported different chromosomes with high occurrences of CNAs, and neither specified if these alterations were gains or losses. While Wils et al. did not find significant associations between the presence of CNAs and progression, Li et al. and Kil et al. reported a significantly higher proportion of samples with CNAs and CNVs in the progressive group over the non-progressive lesions. A full description of the regions reported to have copy number gains and losses is depicted in Table 3.

### 3.3 Gene expression-specific alterations

20 studies evaluated the role of gene alterations in 26 genes in the malignant transformation of OLs (Table 4). With seven studies, the p53 gene family was most commonly investigated (20, 24, 26, 41, 48, 50, 51). While five studies reported p53 overexpression as being associated with malignant transformation (20, 24, 41, 50, 51), two studies evaluating p53 by immunohistochemistry (IHC) did not confirm the associations between p53 expression and malignant transformation (26, 41). Moreover, while Monteiro et al. found no significant relationship between p63 and p73 and malignant transformation, Matsubara et al. reported a significantly higher *Δ*Np63-labelling index in progressed OLs (26, 48).

In addition to p53, the marker genes studied are classified into the following categories (Table 4):

**Cell cycle regulators:** Five marker genes were identified over five studies (20, 22, 24, 26, 47). Increased expression of MKI67, FGFR1 and EZH2 had significant associations with malignant transformation (22, 26, 47). While Qurishi et al. reported that 48.9% of CCND1-positive OLs progressed, no analysis of significance was reported (20). No significant associations between CDKN2A mutations and malignant transformation were found (24, 26).
**Apoptosis regulators:** Three genes over two studies were identified (24, 51). While CASP8 mutations and BAX positivity were more clearly associated with malignant transformation, differences in Bcl-2 immunoreactivity between progressed and non-progressed OLs were not as clearly defined (24, 51).
**Oncogenic signalling pathways:** Four genes over six studies were identified (24, 26, 33, 37, 38, 43). While Monteiro et al. reported no significant relationship between EGFR expression and progression, Ries et al. instead found significantly higher EGFR expression in progressed OLs (26, 43). Although Wils et al. and Ding et al. both reported reduced NOTCH1 expression in OLs with malignant transformation, only Ding et al. found statistical significance (24, 33). Low SMAD4 and high AXIN2 expressions were also significantly associated with malignant transformation (37, 38).
**Cancer stem cell, differentiation and invasion markers:** Six genes were identified over five studies (26, 35, 36, 38, 42). Higher expressions of podoplanin, ALDH1, CD133 and SNAI1 and lower expressions of TGM3 were significantly associated with malignant transformation. No significant relationship was found between CD44 and progression (26).
**Immune/tumour microenvironment:** Three genes were identified over four studies (26, 29, 31, 45). Significantly higher levels of MAGE-A positivity and PTHrP expression were both seen in OLs with malignant transformation (29, 31, 45). No significant relationship was found between BSG/CD147 and progression (26).
**Others:** Wils et al. reported NSD1 mutations to be significantly associated with progression-free survival (24). Liu et al. identified a novel long noncoding RNA, lncRNA oral leukoplakia progressed associated 1 or LOLA1, and reported a significant association between LOLA1 expression and malignant transformation (28).

### 3.4 Proliferative verrucous leukoplakia

Three studies on PVLs investigated the role of DNA ploidy status on malignant transformation risk (Table 5) (23, 40, 49). None of the studies reported significant associations between aneuploidy and malignant transformation. However, Gouvêa et al. and Klanrit et al. did report a high prevalence of aneuploidy in PVLs with malignant transformation (80%–83.3%), compared to those without (50%) in Gouvêa et al's study (40, 49).

Six genes were investigated over three studies for associations with malignant transformation (34, 40, 53). While Chang et al. reported significantly higher progression rates in P53-positive PVLs, Upadhyaya et al. instead found low levels of P53-positivity in PVLs (34, 53). Increased CDKN1A and MCM2 gene expressions were reported to be significantly associated with malignant transformation, while the CDKN2A, MKI67 and GMNN genes were not.

### 3.5 Quality assessment

The risk of bias assessment was performed with the Newcastle-Ottawa Scale for cohort studies for all studies, as shown in Supplementary Table S2. Of the 34 studies included, 19 were found to have a Low risk of bias (7–9 stars), and 15 had an Unclear risk of bias (4–6 stars). None had a High risk of bias (0–3 stars).

## 4 Discussion

This systematic review, encompassing 34 studies with 3,131 oral leukoplakia (OL) patients and 106 patients with proliferative verrucous leukoplakia (PVL), confirms the presence of tissue-based genomic instability markers that are strongly associated with malignant transformation. Specifically, aberrations at the DNA, chromosomal, and gene expression levels appear sequentially or synergistically to drive progression. While no single marker was found to definitively predict malignant transformation, a combination of markers or a progression of instability can be key in identifying lesions with high malignancy risk.

### 4.1 DNA-level and chromosomal instability: the early drivers

Several types of DNA-level and chromosomal alterations were identified to be useful in identifying malignant transformation risk; these alterations often represent early major events that destabilise the genome. DNA ploidy status refers to the number of chromosome sets within a cell, with aneuploidy representing an abnormal number of chromosomes and DNA content. Aneuploidy is a hallmark of cancer that indicates genetic damage that drives carcinogenesis, and is an established prognostic factor in other cancers (54, 55). All studies evaluating DNA ploidy status in this review found significant associations between aneuploidy and malignant transformation. In particular, the high negative predictive value of up to 97.9% may indicate the potential use of DNA ploidy status to rule out a high malignant transformation risk and allow more conservative follow-up regimens for OL patients. However, it is insufficient as a ‘rule-in’ tool for definitive prophylactic wide excision.

The genomic instability indicated by aneuploidy is further compounded by more localised events, such as loss of heterozygosity (LOH). LOH, a mutation resulting in the loss of a copy of a DNA segment, is a common genetic event in carcinogenesis. A LOH event may result in the complete inactivation of essential genes, such as tumour suppressor genes, that favour cancer development. The two studies included in this review evaluating LOH both found significant associations between LOH and malignant transformation of OL. However, no common pattern was found between the two studies; while Graveland et al. identified associations with LOH at chromosome 9p, Jiang et al. found LOH at chromosome 5q, 6p, 6q and 11q instead.

Expanding from localised events like LOH, instability can also occur at the chromosomal level. Chromosomal instability (CIN) refers to alterations in chromosome number and structure during cell division, leading to widespread chromosomal abnormalities such as aneuploidy and LOH. It is a hallmark of cancer, with correlations with tumour stage and metastasis (56). Siebers et al. investigated the role of CIN in malignant transformation of OLs, and reported significant associations between the two, with hazard ratios of 6.8–7.2. The addition of image cytometry of *in-situ* hybridisation analysis for CIN may thus be indicative of high-risk OLs.

Beyond chromosomal alterations, impaired DNA repair mechanisms further exacerbate genomic instability to promote carcinogenesis. Thomas et al. utilised a bleomycin-induced mutagen sensitivity assay (MSA) to quantify the frequency of bleomycin-induced DNA breaks that indicate increased mutagen sensitivity and reduced DNA repair capacity, and reported significantly higher incidence of mutagen hypersensitivity in progressive OLs. The high sensitivity and specificity of 91.7% and 84.0% respectively may indicate its good applicability in differentiating at-risk lesions. Similarly, Farah et al. reported multiple DNA damage repair pathways to be significantly enriched in progressive OLs. This further highlights impaired DNA repair as a contributory factor to carcinogenesis, as defects in DNA repair pathways can lead directly to the accumulation of chromosomal and gene-specific aberrations.

### 4.2 Gene expression-specific alterations: mechanistic correlates

p53, also known as a “guardian of the genome”, is a critical tumour suppressor protein whose mutation is attributed to cancer development. Of the seven studies investigating p53, four reported significant associations between p53 mutation and malignant transformation of OL. Qurishi et al. only reported significant associations between p53, cyclin D1, and microsatellite instability coexpression and progression, while Nogami et al. did not perform any statistical analysis. Identification of p53 mutations by sequencing seems to correlate better with progression; Graveland et al. found significant associations between p53 and progression with sequencing but not immunohistochemistry (IHC), and Monteiro et al., who utilised IHC for detection, reported no significant associations. Therefore, in the consideration of p53 as a genomic marker, while sequencing incurs a higher cost than IHC, clinicians and pathologists should consider sequencing over IHC due to its potentially higher reliability in predicting malignant transformation.

Identifiable cancer stem cell markers may be good indicators of acquired malignant phenotypes that occur after the accumulation of initial genomic damage, characterised by invasion and stemness. Podoplanin and ALDH1 were identified by two studies each in this review to have significant associations with malignant transformation. As both of these markers are detected through IHC, the addition of IHC stains to include markers like podoplanin and ALDH1 may act as a simple risk stratification tool for malignant transformation risk.

While several other genes involved in cell cycle regulation (MKI67, FGFR1, EZH2), apoptosis (CASP8, BAX), oncogenic signalling pathways (NOTCH1, SMAD4, AXIN2), differentiation and invasion (SNAI1, TGM3), and stromal markers (PTHLH) were reported to be significantly associated with malignant transformation, these associations were only reported in one study each and have not yet been validated by other studies. Further studies should be conducted to investigate the applicability of these findings in other patient populations before implementation into clinical practice can be considered.

### 4.3 Contextualisation and clinical correlation

Dysplasia has traditionally been considered one of the key markers for predicting malignant transformation of OL (57). However, even as the current ‘gold standard’, the presence and grade of dysplasia are subjective, inconsistent and have poor specificity. While some studies in this review reported significant associations between dysplasia grade and OL progression, high malignant transformation rates of up to 31.9%–46.7% were still observed in lesions with low-grade dysplasia (22, 31). Furthermore, although initial univariate analyses reported dysplasia to be significantly associated with malignant transformation, multivariate analyses of several studies subsequently either indicated no or marginal associations, and instead found genomic alterations to be more independent predictors of progression (26, 33, 35). Thus, there should be strong consideration to incorporate genomic markers in conjunction with dysplasia for more robust risk evaluation, especially in lesions showing no or mild dysplasia that may, as a result, be falsely determined to be low-risk.

For translation into clinical practice, future research should not focus on identifying a single genomic marker, but on the development and validation of multi-marker genomic panels that combine the most prognostic DNA, chromosomal, and gene expression markers to provide a truly personalised risk assessment. For example, from the findings of this review, a panel combining DNA ploidy status, TP53 and podoplanin or ALDH1 is a strong candidate as a multi-marker panel, where the presence of aneuploidy, TP53 mutation and podoplanin or ALDH1 expression may help to identify a lesion where prophylactic surgery rather than surveillance should be chosen, although this requires prospective validation.

The tissue genomic profiles seen in the patients with PVLs included in this review differ greatly from those of the patients with OLs. No studies found significant associations between aneuploidy and malignant transformation. Similarly, while some gene expressions (MCM2, CDKN1A) were associated with progression, others, including TP53, showed no significant associations. This further reinforces the notion that PVL is a biologically separate entity from OL (58); with higher malignant transformation rates and few consistent clinical and histopathologic predictive factors, the management of PVLs would benefit from more aggressive early surgical intervention.

### 4.4 Strengths and limitations

This systematic review provides a comprehensive search and review of more than 3,200 patients over 34 studies in accordance with the PRISMA guidelines, and is registered on PROSPERO. Previous studies and reviews have focused on clinical predictors, broad biomarker inventories, or individual molecular pathways. In contrast, the present review systematically synthesises tissue-based genomic instability markers evaluated in longitudinal malignant transformation studies and integrates traditional cytogenetic markers with contemporary genomic profiling techniques to inform future biomarker panel development.

However, several limitations need to be discussed. The primary limitation lies in the heterogeneity between included studies. A wide variety of genomic alterations were studied, with few markers discussed across multiple studies. Even among markers evaluated across studies, such as p53, there were significant variations in detection methods and follow-up durations that make comparisons and meta-analyses between studies very challenging. Follow-up duration is also a key confounder for the outcome measure of malignant transformation; this lack of standardisation further limits the validity of the findings. Additionally, as not all samples of each study were successfully analysed for each genomic marker, several studies reported different sample denominators for some genomic markers compared to their reported sample size (20, 30, 39), potentially contributing to a smaller-than-anticipated sample size. Furthermore, all studies included were observational in nature, with no clinical trials. While some studies did perform analyses to account for other known risk factors such as a non-homogeneous appearance and dysplasia, the failure of many studies to control for these covariates limits the ability to determine if a genomic marker is independent of these known clinical predictors. Several included studies also originated from the same institutions or research groups (e.g., Bremmer et al., Graveland et al. and Wils et al.), and thus may have included partially overlapping patient cohorts to evaluate different genomic instability markers. These studies were retained to provide a comprehensive overview of the available literature, but this overlap should be considered when interpreting the strength of the above evidence due to potential for overrepresentation from certain populations. Finally, many gene-specific markers were evaluated in single studies and reported significant associations with malignant transformation, raising the possibility of publication or selective reporting bias, as studies with null findings may be less likely to be published. These findings should therefore be interpreted cautiously and require validation in larger, independent prospective cohorts.

It should also be noted that the malignant transformation rates reported in this review for both OL and PVL are higher than those described in previous studies. The malignant transformation rate of 19.9% for OL and 49.1% for PVL is higher than the 9.8% and 45.8% reported respectively in previous meta-analyses (8, 9). This is because some of the included studies follow a case-control design with the selection of only lesions with malignant transformation for analysis, hence over-representing the number of progressive lesions. Furthermore, for the PVL group, the malignant transformation rate calculated may not be accurate as the majority of samples were from one study (53), while the remaining studies contributed to only a minority of the sample size. More prospective studies with larger cohorts are warranted for the PVL group to obtain the most accurate estimation of its malignant transformation rate.

## 5 Conclusion

This systematic review highlights the key role of genomic alterations at the DNA, chromosomal and gene levels, and demonstrates potential for the use of some tissue-based genomic markers at each level to supplement current clinical and histopathological methods in determining the risk of malignant transformation for OL. While the results are promising, there is still a need for standardisation of detection methods (e.g., with a consensus gene panel or standardised cut-offs) and data reporting (e.g., follow-up duration) to further validate these findings.

## Data Availability

The original contributions presented in the study are included in the article/Supplementary Material, further inquiries can be directed to the corresponding author.
