# Supplementary material for: Tissue-based genomic instability markers for predicting malignant transformation in oral leukoplakia and proliferative verrucous leukoplakia: a systematic review
Source: Front Oral Health. 2026 Aug 25;7:1939172. doi: 10.3389/froh.2026.1939172 (PMC13549864; doi:10.3389/froh.2026.1939172)
Supplement: Supplementary file 1 [file Supplementaryfile1.docx]

**Supplementary Table S1:** Search terms used.

|  | **Search Terms** | | | |
| --- | --- | --- | --- | --- |
| #1 | (“leukoplakia” AND “oral”) | | | |
| #2 | ("transformation" OR "cancer progression" OR "cancerisation" OR "cancerization" OR "progression" OR "squamous cell carcinoma" OR "cancer risk" OR "tumor risk" OR "tumour risk") | | | |
| #3 | (“gene” OR “genetic” OR “genomic” OR “mutation” OR "copy number" OR "gain" OR "amplification" OR “deletion”) | | | |
| Search | #1 AND #2 AND #3 | | | |
| Hits | Search Date: 3 September 2024  Date Limit: No limit – 3 Sep 2024 | Search Date: 27 September 2025  Date Limit: 4 Sep 2024 – 27 Sep 2025 | Search Date: 1 August 2026  Date Limit: 28 Sep 2025 – 1 Aug 2026 | **Total** |
|  | PubMed: 758  Embase: 882  Cochrane: 18 | PubMed: 29  Embase: 78 Cochrane: 1 | PubMed: 24  Embase: 65  Cochrane: 0 | **PubMed: 811**  **Embase: 1025**  **Cochrane: 19**  **Total: 1855** |

**Supplementary Table S2:** Risk of bias assessment with the Newcastle-Ottawa Scale.

| **Study** | **Selection** | | | | **Comparability** | | **Outcome** | | | **Total** |
| --- | --- | --- | --- | --- | --- | --- | --- | --- | --- | --- |
|  | Represent-ativeness | Selection of control | Ascertainment of exposure | Outcome of interest not present at the start | Main factor | Additional factors | Assessment of outcome | Sufficient follow-up | Adequacy of follow-up | (9/9) |
| Qurishi et al | ★ | ★ | ★ | ★ | 0 | 0 | 0 | ★ | ★ | 6/9 |
| Cai et al | ★ | ★ | ★ | ★ | ★ | 0 | 0 | ★ | ★ | 7/9 |
| Mariz et al | ★ | ★ | ★ | ★ | 0 | 0 | 0 | 0 | ★ | 5/9 |
| Pentenero et al | ★ | ★ | ★ | ★ | ★ | 0 | ★ | ★ | ★ | 8/9 |
| Wils et al | ★ | ★ | ★ | ★ | 0 | 0 | ★ | ★ | ★ | 7/9 |
| Jäwert et al | ★ | ★ | ★ | ★ | 0 | ★ | ★ | ★ | ★ | 8/9 |
| Monteiro et al | ★ | 0 | ★ | ★ | ★ | ★ | 0 | 0 | ★ | 6/9 |
| Li et al | ★ | ★ | ★ | ★ | 0 | 0 | 0 | 0 | ★ | 5/9 |
| Liu et al | ★ | ★ | ★ | ★ | ★ | 0 | 0 | ★ | ★ | 7/9 |
| Lv et al | ★ | ★ | ★ | ★ | ★ | ★ | 0 | 0 | ★ | 7/9 |
| Thomas et al | ★ | ★ | ★ | ★ | 0 | 0 | 0 | 0 | ★ | 5/9 |
| Baran et al | ★ | ★ | ★ | ★ | ★ | 0 | ★ | ★ | ★ | 8/9 |
| Farah et al | ★ | ★ | ★ | ★ | 0 | 0 | ★ | ★ | ★ | 7/9 |
| Ding et al | ★ | ★ | ★ | ★ | ★ | ★ | ★ | ★ | ★ | 9/9 |
| Upadhyaya et al | ★ | 0 | ★ | ★ | 0 | 0 | 0 | ★ | ★ | 5/9 |
| Wu et al | ★ | ★ | ★ | ★ | ★ | 0 | ★ | ★ | ★ | 8/9 |
| Habiba et al | ★ | ★ | ★ | ★ | ★ | ★ | ★ | ★ | ★ | 9/9 |
| Sakata et al | ★ | ★ | ★ | ★ | 0 | 0 | 0 | ★ | ★ | 6/9 |
| Zhang et al | ★ | ★ | ★ | ★ | 0 | 0 | 0 | ★ | ★ | 6/9 |
| Kil et al | ★ | ★ | ★ | 0 | 0 | 0 | ★ | ★ | ★ | 6/9 |
| Gouvêa et al | ★ | ★ | ★ | ★ | ★ | 0 | ★ | ★ | ★ | 8/9 |
| Graveland et al | ★ | ★ | ★ | ★ | ★ | 0 | 0 | ★ | ★ | 7/9 |
| Liu et al | ★ | 0 | ★ | ★ | ★ | ★ | 0 | ★ | ★ | 7/9 |
| Ries et al | ★ | ★ | ★ | 0 | ★ | 0 | 0 | ★ | ★ | 6/9 |
| Siebers et al | ★ | 0 | ★ | ★ | 0 | ★ | 0 | 0 | ★ | 5/9 |
| Ries et al | ★ | ★ | ★ | 0 | ★ | 0 | 0 | ★ | ★ | 6/9 |
| Bremmer et al | ★ | 0 | ★ | ★ | ★ | ★ | 0 | ★ | ★ | 7/9 |
| Cao et al | ★ | 0 | ★ | ★ | ★ | ★ | 0 | ★ | ★ | 7/9 |
| Matsubara et al | ★ | ★ | ★ | ★ | ★ | 0 | 0 | ★ | ★ | 7/9 |
| Klanrit et al | ★ | ★ | ★ | 0 | 0 | 0 | 0 | ★ | ★ | 5/9 |
| Mogi et al | ★ | ★ | ★ | ★ | ★ | 0 | 0 | ★ | ★ | 7/9 |
| Nogami et al | ★ | ★ | ★ | ★ | 0 | 0 | 0 | ★ | ★ | 6/9 |
| Jiang et al | ★ | 0 | ★ | 0 | 0 | 0 | ★ | ★ | 0 | 4/9 |
| Chang et al | ★ | ★ | ★ | ★ | ★ | ★ | 0 | ★ | ★ | 8/9 |
